# Breast–Ovarian Hereditary Cancer Syndrome: Beyond *BRCA1* and *BRCA2*

**DOI:** 10.3390/curroncol33090556

**Published:** 2026-09-14

**Authors:** Evgeny Imyanitov, Anna Sokolenko

**Affiliations:** 1 Department of Tumor Growth Biology, N.N. Petrov Institute of Oncology, 197758 Saint-Petersburg, Russia; 2 Department of Medical Genetics, St.-Petersburg State Pediatric Medical University, 194100 Saint-Petersburg, Russia

**Keywords:** breast cancer, germline pathogenic variant, NCCN guidelines, non-BRCA genes, ovarian cancer

## Abstract

Although *BRCA1* and *BRCA2* remain the backbone for germline DNA testing of patients with breast cancer (BC) or ovarian cancer (OC), there are a number of other genes with proven or potential clinical significance. *PALB2* is associated with an elevated risk of BC, whereas pathogenic variants (PVs) in *RAD51C*, *RAD51D*, and *BRIP1* are mainly relevant to OC development. Some germline findings are helpful in guiding therapeutic decisions: for example, *PALB2*, *RAD51C*, and *RAD51D* germline PVs render tumors sensitive to PARP inhibitors (PARPi), whereas cancers arising in *PTEN* heterozygotes are likely to be responsive to AKT down-regulators. *CHEK2*, *ATM*, *BLM*, and *NBN* PVs result in only a two-fold or even lower excess of cancer risk. However, the incorporation of these genes in DNA testing panels may occasionally lead to the identification of individuals with biallelic germline inactivation; these patients have a severe disease phenotype and thus require intensive medical intervention. The accumulation of data on “non-*BRCA*” BC- and OC-predisposing genes is complicated due to the rarity of their alterations, significant interethnic variations in population frequency of relevant PVs, heterogeneity of disease subtypes, etc. The use of extended gene panels, which pool together both clinically validated cancer-associated genes and “candidate” genes with potential but unproven significance, is likely to be a prevailing diagnostic approach in the next few years; therefore, proper attitudes towards accumulation and interpretation of genetic data are important.

## Simple Summary

Women with breast cancer (BC) or ovarian cancer (OC) routinely receive germline DNA testing for diagnosis of hereditary cancer syndromes. This testing was confined to *BRCA1* and *BRCA2* genes in the past, however, current technologies allow simultaneous analysis of a number of additional genes for the same cost. None of the “novel” BC/OC-predisposing genes are fully identical to *BRCA1*/*2* with respect to the clinical significance. There are substantial gene-specific differences regarding the spectrum of associated tumor types, the degree of elevation of cancer risks, and the optimal choice of anticancer drugs. This review provides an update on the BC/OC-predisposing role of “non-*BRCA*” genes, with the emphasis on existing controversies, knowledge gaps, and further research activities.

## 1. Introduction

*BRCA1* and *BRCA2* genes were discovered in the mid-1990s via analysis of large families with multiple instances of breast and ovarian cancers. It was immediately recognized that *BRCA1* and *BRCA2* explain only a subset of patients with clinical signs of hereditary breast–ovarian cancer (HBOC) syndrome; therefore, it looked apparent at that time that the discovery of similarly significant contributors would become a part of routine research activities. Despite these expectations, neither the analysis of *BRCA1*/*2*-negative families nor comprehensive case-control studies led to the discovery of “*BRCA3*”. Although a number of high-risk genes with penetrance similar to *BRCA1*/*2* have been identified, their contribution to breast or ovarian cancer morbidity seems limited due to very low population frequency of the corresponding pathogenic variants (PVs). In addition, there are a couple of genes characterized by a significant frequency of PVs but only a moderate excess of cancer predisposition. A comprehensive analysis of “non-*BRCA*” breast–ovarian cancer-predisposing genes is a subject of continuous updates in the NCCN guidelines [1]. Practical attitudes towards the most commonly tested genes have recently been discussed in recommendations issued by the ESMO Precision Oncology Working Group [2]. This review focuses on controversies and knowledge gaps related to the role of “non-*BRCA*” PVs (Table 1 and Table 2).

## 2. Genes with Overt Clinical Significance

Several dozen genes have emerged as potential causes of HBOC; however, only a limited number of candidates (*PALB2*, *RAD51C*, *RAD51D*, *BRIP1*, and *TP53*) have demonstrated sufficiently strong and reproducible associations with either BC or OC risk to warrant medical intervention. In addition, alterations in some genes discussed below (*PALB2*, *RAD51C*, *RAD51D*, and *PTEN*) call for the administration of targeted therapy. Overall, the accumulation of data for highly penetrant “non-*BRCA*” contributors turned out to be a challenge due to the rarity of corresponding PVs and significant interstudy variations.

### 2.1. PALB2

*PALB2* is the most established BC-predisposing gene after *BRCA1* and *BRCA2*. Its penetrance towards BC is comparable to that of *BRCA1*/*2* [18,19]. The contribution of *PALB2* PVs to BC morbidity varies between studies, with most datasets placing this gene in the third position after *BRCA1* and *BRCA2* [18,19]. The association between *PALB2* and OC risk is at best moderate [3,56]. However, current NCCN guidelines for *PALB2* PV carriers recommend prophylactic surgery both for breasts and ovaries, apparently reflecting the low efficacy of early OC detection coupled with no impact of risk-reducing salpingo-oophorectomy (RRSO) on the quality of life in women aged over 50 years. *PALB2*-driven BCs show a high rate of somatic inactivation of the wild-type allele; this results in homologous recombination deficiency (HRD) and, consequently, high tumor sensitivity to platinum compounds and PARP inhibitors (PARPi) [4,39,40,57]. Similar data have been obtained in ovarian and prostate cancer studies [5,58,59]. Some investigations described a significant frequency of gross deletions or duplications in the *PALB2* gene, which may not be detectable by conventional next-generation sequencing (NGS) [57].

### 2.2. RAD51C and RAD51D

The magnitude of predisposition to OC in carriers of *RAD51C* or *RAD51D* PVs is similar to that observed in *BRCA2* heterozygotes; therefore, RRSO is recommended as an option. In contrast, these genes render only a two-fold elevation of BC risk, thus dismissing the feasibility of risk-reducing mastectomy (RRM) [12]. The majority of *RAD51C*- and *RAD51D*-driven OCs have an HRD phenotype associated with tumor sensitivity to PARPi and platinum compounds [5,8,42]. Loss of the remaining allele and HRD are observed only in triple-negative *RAD51C*/*D*-driven BCs, whereas estrogen receptor-positive breast tumors retain the wild-type copy of the involved gene and demonstrate homologous recombination proficiency [42].

### 2.3. BRIP1

*BRIP1* is apparently the only example of a gene that shows a reproducible association with OC risk but does not increase susceptibility to BC [13]. The lifetime probability of OC development in *BRIP1* PV carriers is within 5–15%, which is sufficient to consider RRSO according to the NCCN recommendations [1,12]. Somatic inactivation of the remaining allele is common in *BRIP1*-associated OCs [5]. Surprisingly, despite several OC datasets reporting instances of *BRIP1*-driven carcinomas [5,14], neither HRD status nor platinum sensitivity have been assessed in this tumor subset. Preclinical data demonstrate increased sensitivity of *BRIP1*-deficient cells to PARPi [45].

### 2.4. TP53

*TP53* is the only “syndromic” gene recommended for inclusion in BC diagnostic gene panels by the ESMO Precision Oncology Working Group [2]. *TP53* PVs are particularly common in very young-onset BCs, although occasional findings occur across all age groups. Management of *TP53* PV carriers is a complex task, given the multiorgan cancer risks associated with this syndrome [46]. The NCCN guidelines suggest considering RRM [1]. Current recommendations do not account for the variable penetrance of distinct *TP53* PVs, which seems to be a significant drawback [60]. Approximately half of *TP53*-associated BCs are HER2-positive, which is about twice as high as in unselected BC patients [16,47,48]. It appears that the presence of *TP53* germline PVs does not compromise the efficacy of HER2-directed therapies [48].

### 2.5. PTEN

The ESMO Precision Oncology Working Group did not recommend including *PTEN*, a gene associated with Cowden/hamartoma tumor syndrome, in routine BC germline DNA testing; this position is attributed to the rarity of *PTEN* PVs and uncertainty in clinical decisions in patients with chance findings of inherited *PTEN* alterations [2]. Although some case-control series showed only a moderate enrichment of BC patients by *PTEN* PVs [18,19], the NCCN guidelines mention the high risk of BC in *PTEN* PV carriers and suggest consideration of RRM [1].

Technical aspects of *PTEN* genetic testing deserve particular consideration. Among non-BRCA genes, *PTEN* is the best-documented example of germline promoter PVs, which cause a fully penetrant syndromic phenotype. These PVs result in an abnormal composition of 5′-end untranslated mRNA that eventually affects protein translation [61]. Many of the currently utilized multigene NGS panels and bioinformatics pipelines are limited to the analysis of coding exons; therefore, they may miss clinically relevant alterations in promoter regions.

The utility of *PTEN* testing needs to be reconsidered in light of the recent clinical approval of the AKT inhibitor capivasertib. Kingston et al. [49] demonstrated exceptional responses in tumors associated with germline *PTEN* PVs. Although the frequency of *PTEN* PVs in unselected BC patients is below 1:3000 [18,19], the availability of relevant drugs may warrant routine *PTEN* analysis.

## 3. Moderate-Penetrance Genes and Genes with Disputed Clinical Significance

There are a number of genes (*ATM*, *CHEK2*, *BARD1*, *NBN*, *BLM*, etc.) whose heterozygous inactivation is associated with an approximately two-fold elevation in BC risk. Although they are all involved in the repair of DNA double-strand breaks, the associated tumors usually do not have HRD and, therefore, are insensitive to PARPi. Given the uncertainty of the actual survival benefits of intensive surveillance, lack of indications for prophylactic surgery, and the absence of associated therapies, the ESMO Precision Oncology Working Group recommends against the inclusion of low-penetrance genes in BC testing panels [2]. This position somehow contradicts the reality, given that at least *ATM* and *CHEK2* are included in all clinically approved BC germline DNA tests [2]; therefore, in the near future, doctors and patients will continue to receive the results of *ATM*, *CHEK2*, etc., testing.

While relevant information on a gene-by-gene basis is presented in Table 1 and Table 2, we discuss below some nuances common for the entire group of these genes.

### 3.1. Chance Findings of Subjects with Biallelic Germline PVs

The population frequency of protein-truncating PVs in the *CHEK2* gene approaches 1% in some European countries. Not surprisingly, several dozen patients with biallelic *CHEK2* inactivation have been reported in the literature. Many of these individuals are described as having multiple tumors; thus, the severity of the biallelic *CHEK2* PV phenotype may resemble Li–Fraumeni syndrome [62,63]. Strikingly, cytogenetic analysis of lymphocytes obtained from subjects with *CHEK2* germline inactivation demonstrated multiple chromosomal abnormalities [64]. Although guidelines for the clinical management of these individuals have not yet been established, it is self-explanatory that patients with biallelic *CHEK2* protein-truncating PVs need to be detected and tightly monitored. The BC-predisposing roles of *BLM* and *NBN* have been suggested in some, although not all, studies. Both genes cause severe immune deficiency when inactivated in the germline (Bloom syndrome and Nijmegen breakage syndrome, respectively). Until recently, both these conditions were believed to have overt clinical manifestations starting in early childhood and, therefore, appeared easily recognizable. Recent data obtained from routine DNA germline testing have revealed unexpected instances of these potentially fatal syndromes in otherwise seemingly healthy adult cancer patients [65,66]. Biallelic germline *ATM* inactivation is associated with ataxia–telangiectasia [67]; it remains to be seen whether mild forms of this syndrome will ever be detected upon BC genetic testing. Overall, the instances described above may justify the retention of *ATM*, *CHEK2*, *BLM*, and *NBN* in NGS panels utilized for the diagnosis of hereditary cancer syndromes.

### 3.2. Ethnic Aspects

A high occurrence of *BLM* PVs is observed mainly in Ashkenazi Jews and Slavic people, and *NBN* germline PVs are described almost exclusively in countries with predominantly Slavic populations [68,69]. This ethnic specificity precludes global validation of the observed cancer-predisposing effects. There will be a growing number of genes whose potential significance is demonstrated in a particular founder population, but replication of the observed effects is complicated due to low population frequency of relevant gene alterations in other ethnic groups. This problem deserves to be addressed; otherwise, ethnicity-specific medically important PVs are likely to be missed.

### 3.3. Optimal Composition of Gene Panels

Many diagnostic gene panels include loci with suspected but unproven BC/OC-predisposing significance (*BLM*, *NBN*, *FANCC*, *FANCM*, *MRE11*, *RAD50*, *RECQL*, etc.). In addition, there is a growing tendency to replace organ-specific genetic tests with “universal” DNA assays covering all known hereditary cancer genes [2]. Although extended testing significantly increases the probability of chance findings of uncertain clinical actionability, it is likely to become a prevailing diagnostic attitude owing to convenience and cost efficiency. While it is self-explanatory that gene panels utilized for medical purposes must contain only well-validated contributors to cancer susceptibility, the practical implementation of this principle appears complicated. In fact, even extraordinarily large case-control studies sometimes produce spurious findings. For example, recent gene burden analysis involving 29,535 BC patients and 374,104 controls showed lower penetrance for *BRCA2* than for *ATM* or *CHEK2* [70]. Many well-established BC-associated genes predispose only to a particular subtype of this disease, e.g., triple-negative or hormone receptor-positive BC, which further complicates the estimation of overall disease-associated risks [23,42]. In this respect, the accumulation of real-world data through routine germline DNA testing is a viable approach to finalize the list of medically relevant cancer-predisposing genes and to establish appropriate guidelines for the clinical management of PV carriers. Perhaps the problem lies not in the composition of BC/OC gene panels per se, but in the interpretation of findings involving “candidate” or “syndromic” genes.

## 4. Lynch Syndrome Genes

Lynch syndrome is a common hereditary disease, which manifests by highly elevated predisposition to gastrointestinal and endometrial malignancies. It is caused by germline PVs in DNA mismatch repair (MMR) genes. Tumors arising due to somatic MMR inactivation accumulate multiple mutations and exert significant sensitivity to inhibitors of immune checkpoints. Women with Lynch syndrome have increased likelihood of OC development. *MSH2* and *MLH1* genes show the strongest and the most consistent associations with elevated OC risk. This risk may be somewhat lower for *MSH6* PV carriers, while the data for *PMS2* remain inconclusive [71]. Lynch-associated OC has a distinctive histologic presentation: it is enriched for endometrioid and clear cell carcinomas, with high-grade serous tumors being relatively uncommon [54]. Women with confirmed Lynch syndrome, particularly due to *MSH2* or *MLH1* PVs, are often suggested to consider risk-reducing salpingo-oophorectomy (typically together with hysterectomy) after completion of childbearing, while recommendations for *MSH6* and *PMS2* carriers are more individualized given their lower absolute OC risks [72].

## 5. Future Research Directions

### 5.1. Many Clinically Relevant Genes Predispose Only to Particular Subtypes of BC or OC

Preference towards particular tumor subtypes was acknowledged soon after the discovery of the BC- and OC-predisposing role of *BRCA1* and *BRCA2* genes. It is well established that while *BRCA1* is associated mainly with triple-negative disease and tends to be particularly overrepresented in young-onset patients, *BRCA2* PV carriers often develop hormone-receptor-positive disease in their fifties or sixties [43]. Similarly, both *BRCA1* and *BRCA2* are linked not to OC in general, but to its particular histological subtype, i.e., high-grade serous OC (HGSOC) [73]. These nuances did not compromise the discovery of *BRCA1* and *BRCA2.* Indeed, both these genes combine high penetrance and noticeable population frequency. Therefore, presence of potentially irrelevant categories of patients, such as elderly women with receptor-positive BC in *BRCA1* analysis or non-HGSOC in OC studies *BRCA1*/*2,* may result in some underestimation of associated risks, but not in the entire collapse of gene–disease associations.

Genes with moderate penetrance or vanishingly low population frequencies are more vulnerable to drawbacks in patient selection. For example, *ATM* and *CHEK2* PVs are associated mainly with the risk of estrogen-receptor-positive BC; consequently, consideration of this BC subtype as a separate disease entity results in some increase of *ATM-* and *CHEK2*-associated risks [19,74]. This enrichment does not translate into overt clinical relevance of *ATM* and *CHEK2* because hormone-receptor-positive BC is the most common disease variety. However, the situation becomes more dramatic if a given gene renders the increased risk for triple-negative (basal-like) BC, but not for other BC categories. Triple-negative BCs usually constitute only 15–30% of unselected BC patients; therefore, the “dilution effect” may indeed be critical. This factor is essential, for example, for *BARD1*, *RAD51C*, and *RAD51D*, which demonstrate moderate risks for BC overall but appear to be high-penetrance genes for estrogen-receptor-negative and triple-negative disease [18]. Similar associations have been reported for *FANCM*, whose protein-truncating variants are also preferentially linked to triple-negative BC [75,76]. *CDH1* is an even more illustrative example of the role of BC histological classification: its germline loss-of-function variants confer a cumulative BC risk of approximately 37% in female carriers, but this genetic predisposition is essentially restricted to invasive lobular carcinoma, a subtype comprising no more than 5–15% of BC overall [77]. Not surprisingly, rough comparisons of *CDH1* PV frequencies in unselected BC patients versus controls make its impact invisible, while lobular-enriched BC cohorts easily recover its role [78].

The significance of the disease subtype is even more pronounced in OC studies. As already mentioned above, *BRCA1*/*2* are irrelevant to non-HGSOC histologies, which account for approximately one third of OC incidence [79]. Conversely, Lynch syndrome genes, particularly *MSH6*, and the recently identified *HELB*, are linked to non-HGSOC predisposition but do not show elevated frequency of PVs in HGSOCs [80,81,82].

The potential of genomic studies involving unselected BC or OC patients appears to already be exhausted [70]. However, proper stratification of cancer patients—for example, separate consideration of women with young-onset versus late-onset disease, hormone-receptor-positive versus triple-negative versus *HER2*-amplified BC, HGSOC versus non-HGSOC, or HRD versus non-HRD cancers, is likely to reveal previously unrecognized gene–disease associations or, at minimum, clarify the impact of genes with disputed cancer-predisposing significance.

### 5.2. Emphasis on Ethnicity-Adjusted Analysis and Yet Unstudied Ethnic Groups

Even early *BRCA1*/*2* studies revealed that ethnic roots play an essential role in gene–disease associations. For example, patients of Slavic descent are characterized by overrepresentation of *BRCA1* PVs but relatively low frequency of *BRCA2* PVs, while the opposite trend is observed in Finland, Iceland, Tuva, etc. [83,84,85,86]. The study of several large but relatively autonomous ethnic communities residing in the Caucasus mountains demonstrated that although the overall impact of *BRCA1*/*2* on BC and OC morbidity was more or less similar across different regions, the ratio between *BRCA1* and *BRCA2* PV carriers showed striking population-specific variations [87]. While virtually all countries studied to date confirm the role of either *BRCA1*, *BRCA2*, or both, the effects of other potentially significant genes are very likely to be diluted when populations with variable background frequencies of gene-specific PVs are pooled together.

Two complementary mainstream approaches deserve discussion. First, reanalysis of existing data sets with proper adjustment for ethnicity may specify the associated risks for recently discovered cancer-predisposing genes and reveal significant players among suspected gene candidates. Secondly, genomic investigations involving BC and OC patients from yet unstudied ethnic groups, particularly from communities with significant genetic homogeneity, have to be encouraged. In particular, members of *RAD* and *FANC* gene families deserve comprehensive studies. This is a significant challenge, given that many potentially interesting autochthonous human communities may be poorly accessible due to the substandard level of BC and OC diagnosis and registration, difficulties associated with the proper collection and characterization of biological samples, and many other obstacles. Still, these barriers can realistically be overcome with reasonable support from research grants.

### 5.3. Gross Genetic Alterations in HBOC Genes

*BRCA1*/*2* genes were initially studied by Sanger sequencing. However, this method is unable to detect deletions or duplications of entire exons, which are described in literature as so-called large gene rearrangements (LGRs) or copy number variations (CNVs). These gross abnormalities can be detected by MLPA (multiplex ligation-dependent probe amplification), and this method was included in *BRCA1*/*2* testing guidelines for all patients, who appeared PV-negative upon Sanger sequencing [88]. Consequently, the impact of LGRs in *BRCA1*/*2* PV distribution is described in great detail, and it is known that they contribute to a substantial proportion of *BRCA1*/*2*-related morbidity [89]. *CHEK2* is another relevant example: a gross 5395-bp deletion involving exons 9–10 (del5395) represents a major share of protein-truncating *CHEK2* PVs, particularly in countries with predominantly Slavic populations [90]. However, the contemporary approach to DNA germline analysis almost entirely relies on NGS. NGS pipelines are rarely adjusted to the detection of LGRs [91]. Consideration of this category of PVs is essential for the unbiased analysis of recently discovered BC- and OC-predisposing genes.

### 5.4. Autosomal-Recessive Mechanism of Inheritance?

The majority of hereditary diseases revealed by medical genetic studies have an autosomal recessive mechanism of inheritance. This is attributed to the nature of medical genetic investigations, which usually focus on very rare diseases with exceptionally characteristic phenotypes. Consequently, the majority of these patients have unique features allowing for clear-cut discrimination between cases and non-affected subjects. The instances of autosomal recessive inheritance are known in cancer genetics, being applicable to patients with well-defined and peculiar disease presentation (for example, *MUTYH*-associated polyposis, xeroderma pigmentosum, or Bloom syndrome [68,92,93]).

The search for recessive causes of common cancers, such as breast, lung, or colorectal malignancies, is very complicated, given that the tumors with the same appearance commonly arise sporadically. Consequently, while pedigrees with two–three instances of uncommon disease attract medical genetic investigations, large families with two–three cases of, for example, BC, do not look promising in the search for recessive PVs. Systematic exome studies on patients with family-history-negative bilateral BC are probably promising in this respect [94].

### 5.5. Therapeutic Implications

The emergence of gene-specific drugs boosts interest in tumors carrying respective genetic alterations. This is relevant to DNA germline PVs, irrespective of whether the associated penetrance is high or moderate.

As already mentioned above, several non-*BRCA1*/*2* genes, particularly *BRIP1* and *BARD1*, deserve to be analyzed for HRD status in the associated tumors, thus clarifying whether PARPi can be effectively utilized in this category of cancer patients. Furthermore, the attitudes towards administration of PARPi or platinum compounds in carriers of PVs in *PALB2*, *RAD51C*, *RAD51D*, etc., genes need to be established. In *BRCA1*/*2* PV carriers, the mere detection of a germline variant in either of these genes is currently considered sufficient for the choice of targeted therapy [95]. This is an oversimplification, given that even *BRCA1*/*2*-driven cancers do not always demonstrate somatic inactivation of the remaining allele of the involved gene [96]. The situation appears to be somewhat more complex for “novel” HBOC-predisposing genes, where significant variability of the “second-hit” status is observed [5]. Perhaps administration of targeted therapy solely on the basis of germline DNA testing has to be discouraged, given that the analysis of somatic inactivation of the wild-type allele of the involved gene, preferably coupled with the HRD evaluation, may significantly improve precision in the prediction of drug response.

Clinical studies have revealed instances of tumor response to the ATR inhibitor camonsertib in *ATM* PV heterozygotes [97]. The analysis of lobular BCs, which are common among *CDH1* PV carriers, led to discovery of synthetic lethal interactions between E-cadherin loss and ROS1 inhibition. This translated into a neoadjuvant clinical trial evaluating the efficacy of combining conventional endocrine therapy with entrectinib [98]. Clinical information on *PTEN*-associated BCs treated by capivasertib or similar drugs has to be collected in a systematic way to accumulate relevant real-world evidence [49].

## 6. Conclusions

The invention of NGS has dramatically changed attitudes towards germline DNA testing, with a growing number of cancer patients and healthy individuals undergoing multigene, whole-exome, or whole-genome sequencing. The accumulation of large datasets has mainly resulted in the downgrading of penetrance estimates for some genes previously regarded as significant contributors to cancer risk, while, surprisingly, it did not really result in the discovery of novel HBOC-predisposing loci. Although the majority of genes included in diagnostic panels and clinical guidelines have been known for many years, the information on associated cancer risks, optimal management of healthy PV carriers, and tumor drug sensitivity remains insufficient. It is important to encourage researchers, practicing oncologists, and DNA diagnostic services to collect unbiased data on PV frequencies in cancer patients and controls, gene-associated tumor phenotypes, and treatment results. It seems likely that the majority of high- and moderate-penetrance autosomal-dominant genes causing BC or OC predisposition in women of European descent have already been identified. Emphasis on previously unstudied ethnic groups, consideration of missense and non-coding germline alterations, and analysis of autosomal recessive inheritance have the potential to reveal new cancer-predisposing loci.

## Figures and Tables

**Table 1:** Cancer risks in carriers of PVs in BC- and OC-predisposing genes.

Gene	BC Risk/Penetrance (Lifetime Risk)	OC Risk/Penetrance (Lifetime Risk)	Risk of Other Malignancies (Lifetime Risk)	Role in Particular Ethnic Groups	Phenotype of Biallelic PV Carriers	References
Genes with overt clinical significance						
*PALB2*	High (33–58%, depending on family history)	Moderate (3–5%)	Male BC (1%), pancreatic cancer (5–10%), prostate cancer (possibly elevated)		FA	[3,4,5,6,7]
*RAD51C*	Moderate (20–40%)	High (11–13%)	?		FA	[8]
*RAD51D*	Moderate (20–40%)	High (11–13%)	?	Common in some Asian ethnic groups	-	[8,9,10,11]
*BRIP1*	Not increased	High (6–15%)	?		FA	[12,13,14,15]
*TP53*	High (up to 100%)	Not increased	Li-Fraumeni syndrome	*TP53* R337H allele is common in Brazil	-	[16,17]
*PTEN*	High (67–85%)	Not increased	Cowden syndrome		-	[18,19,20,21]
Moderate-penetrance genes and genes with disputed clinical significance						
*CHEK2*	Moderate (20–33%)	Not increased	Multiple cancer types	Common in Northern/Central Europe and Slavic populations	Severe predisposition to multiple cancers	[18,22,23]
*ATM*	Moderate (21–24%)	Moderate or unproven	Multiple cancer types		Ataxia-telangiectasia	[18]
*BLM*	Moderate or unproven	?	?	*BLM* Q548X allele is common Slavic populations	Bloom syndrome	[24,25]
*NBN*	Moderate or unproven	Moderate or unproven	?	*NBN* c.657del allele is common in Slavic populations	Nijmegen breakage syndrome	[26,27]
*BARD1*	Moderate (17–33%)	Not increased	?		-	[18]
*MSH2*	Unproven	High (11%)	Lynch syndrome	Founder alleles are described in several populations (e.g., Portuguese, Ashkenazi Jewish)	CMMRD	[28,29,30,31]
*MLH1*	Unproven	High (17%)	Lynch syndrome	Founder alleles are described in several populations (e.g., Finnish, Swedish)	CMMRD	[28,31,32,33]
*MSH6*	Unproven	Moderate (risk varies across studies from 1 to 13%)	Lynch syndrome	Founder alleles are described in several populations (e.g., Icelandic, Swedish, Ashkenazi Jewish)	CMMRD	[28,31,34,35,36,37]
*PMS2*	Unproven	Unproven or low (<3%)	Lynch syndrome	Founder alleles are described in several populations (e.g., Icelandic, French Canadian)	CMMRD	[28,35,38]

BC—breast cancer; CMMRD—constitutional mismatch repair deficiency; FA—Fanconi anemia; OC—ovarian cancer; PV—pathogenic variant; ? —data incomplete.

**Table 2:** Clinical and molecular features of tumors associated with germline PVs in BC- and OC-predisposing genes.

Gene	Association with Specific BC or OC Features	Feasibility of Intensive Surveillance	Feasibility of Risk-Reducing Surgery	LOH in BC or OC	HRD	Drug Sensitivity	References *
*PALB2*	?	+	+ (RRM), ? (RRSO)	+	+	PARPi, platinum	[39,40,41]
*RAD51C*	Triple-negative BC	+	+ (RRSO)	+	+	PARPi, platinum	[5,42,43]
*RAD51D*	Triple-negative BC	+	+ (RRSO)	+	+	PARPi, platinum	[5,10,42,43,44]
*BRIP1*	-	+	+ (RRSO)	+	?	?	[5,12,45]
*TP53*	Very early-onset; HER2+, often triple-positive BC	+	+ (RRM)	+	-	HER2-directed therapies in HER2+ tumors	[46,47,48]
*PTEN*	?	+	+ (RRM)	+	?	AKT inhibitors	[20,49]
*CHEK2*	Hormone receptor-positive BC	?	-	+/-	-		[22,23]
*ATM*	Hormone receptor-positive BC	?	-	+	-		[43,50,51]
*BLM*	?	?	-	-	-		[25,26]
*NBN*	-	?	-	-	-		[26,52]
*MSH2*	Mostly non-serous (endometrioid or, less often, clear cell) OC	+	+ (RRSO)	+	-	Immune checkpoint inhibitors	[53,54,55]
*MLH1*	Mostly non-serous (endometrioid or, less often, clear cell) OC	+	+ (RRSO)	+	-	Immune checkpoint inhibitors	[53,54,55]
*MSH6*	Mostly non-serous (endometrioid or, less often, clear cell) OC	+	+ (RRSO), individualized	+	-	Immune checkpoint inhibitors	[53,54,55]
*PMS2*	Mostly non-serous (endometrioid or, less often, clear cell) OC	?	-	+	-	Immune checkpoint inhibitors	[53,54,55]

* In addition to references mentioned in Table 2, each gene was considered for information presented in [1,2]. BC—breast cancer; HRD—homologous recombination deficiency; LOH—loss-of-heterozygosity; OC—ovarian cancer; PARPi—poly (ADP-ribose) polymerase inhibitor; RRM—risk-reducing mastectomy; RRSO—risk-reducing salpingo-oophorectomy; ? —data incomplete; +/-—present in a subset of cases.

## Data Availability

No new data were created or analyzed in this study. Data sharing is not applicable to this article.

## Abbreviations

The following abbreviations are used in this manuscript:

BC	Breast cancer
FA	Fanconi anemia
HBOC	Hereditary breast–ovarian cancer
HRD	Homologous recombination deficiency
LGR	Large gene rearrangement
LOH	Loss-of-heterozygosity
MMR	Mismatch repair
NGS	Next-generation sequencing
OC	Ovarian cancer
PARPi	Poly (ADP-ribose) polymerase inhibitor
PV	Pathogenic variant
RRM	Risk-reducing mastectomy
RRSO	Risk-reducing salpingo-oophorectomy
